# Selenium speciation studies in cancer patients to evaluate the responses of biomarkers of selenium status to different selenium compounds

**DOI:** 10.1007/s00216-024-05141-y

**Published:** 2024-01-30

**Authors:** M. Estela del Castillo Busto, Christian Ward-Deitrich, Stephen O. Evans, Margaret P. Rayman, Michael B. Jameson, Heidi Goenaga-Infante

**Affiliations:** 1 LGC Limited, National Measurement Laboratory (NML), Queens Road, Teddington, Middlesex TW11 0LY UK; 2 Grupo Química Analítica Aplicada (QANAP), Instituto Universitario de Medio Ambiente (IUMA), Universidade da Coruña (UDC), 15071 A Coruña, Spain; 3 Department of Biological Sciences, University of Waikato, Hamilton, New Zealand; 4 Waikato Clinical Campus, University of Auckland, Hamilton, New Zealand; 5 Department of Nutritional Sciences, University of Surrey, Guildford, GU2 7XH UK; 6 Oncology Department, Waikato Hospital, Hamilton, New Zealand

**Keywords:** Selenium speciation, Sodium selenite, Seleno-L-methionine, Se-methylselenocysteine, Clinical trial, ICP-MS

## Abstract

**Abstract:**

This work presents the first systematic comparison of selenium (Se) speciation in plasma from cancer patients treated orally with three Se compounds (sodium selenite, SS; L-selenomethionine, SeMet; or Se-methylselenocysteine, MSC) at 400 µg/day for 28 days. The primary goal was to investigate how these chemical forms of Se affect the plasma Se distribution, aiming to identify the most effective Se compound for optimal selenoprotein expression. This was achieved using methodology based on HPLC-ICP-MS after sample preparation/fractionation approaches. Measurements of total Se in plasma samples collected before and after 4 weeks of treatment showed that median total Se levels increased significantly from 89.6 to 126.4 µg kg^−1^ Se (*p* < 0.001), particularly when SeMet was administered (190.4 µg kg^−1^ Se). Speciation studies showed that the most critical differences between treated and baseline samples were seen for selenoprotein P (SELENOP) and selenoalbumin after administration with MSC (*p* = 5.8 × 10^−4^) and SeMet (*p* = 6.8 × 10^−5^), respectively. Notably, selenosugar-1 was detected in all low-molecular-weight plasma fractions following treatment, particularly with MSC. Two different chromatographic approaches and spiking experiments demonstrated that about 45% of that increase in SELENOP levels (to ~ 8.8 mg L^−1^) with SeMet is likely due to the non-specific incorporation of SeMet into the SELENOP affinity fraction. To the authors’ knowledge, this has not been reported to date. Therefore, SELENOP is probably part of both the regulated (55%) and non-regulated (45%) Se pools after SeMet administration, whereas SS and MSC mainly contribute to the regulated one.

**Graphical abstract:**

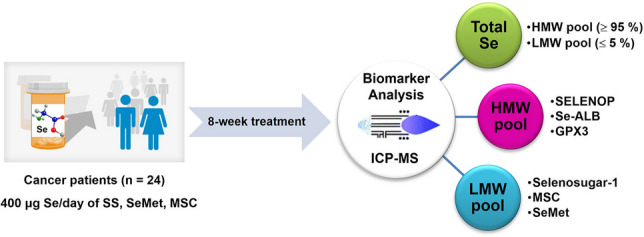

**Supplementary information:**

The online version contains supplementary material available at 10.1007/s00216-024-05141-y.

## Introduction

Selenium (Se) is an essential trace element often used as a supplement for the prevention or control of cancer due to its history in these areas [1]. Randomised controlled trials of Se supplementation for cancer prevention have shown conflicting results, highlighting the complexity of Se metabolism owing to the various toxicological and physiological properties of Se species [2]. Notably, in 1996, the Nutritional Prevention of Cancer (NPC) trial [3] demonstrated reduced cancer incidence (prostate, colon, lung) and mortality in US patients with a previous history of non-melanoma skin cancer (1312 volunteers) after 7.4 years of daily supplementation with 200 µg Se per as selenised yeast. In contrast, the Selenium and Vitamin E Cancer Prevention Trial (SELECT) [4] showed no protection of 35,533 Se-replete men against prostate and other major cancers when the same dose was used. However, in SELECT, L-selenomethionine (SeMet), alone or in combination with vitamin E, was administered for 5.5 years. The divergent outcomes emphasise the importance of the specific administered Se form (species) and the Se status of the targeted population. The evidence suggests that Se supplementation would only be beneficial for populations with low or relatively low serum/plasma Se status, such as those in the NPC Trial (mean plasma Se 114 µg/L Se), while those with adequate or high Se status (≥ 122 µg/L Se) should not be supplemented; the plasma Se for the entire SELECT cohort exceeded this level, with mean ~ 135 µg/L Se) [1]. Furthermore, these trials used different sources of Se: SeMet (in SELECT) *vs* selenised yeast (in NPC); the latter contains SeMet (~ 35%) among 15 other species [5, 6].

Previous findings relate to cancer prevention studies wherein Se supplement doses remained below the tolerable upper intake level (UL) (255 µg/day) [7]. Nonetheless, there have been separate studies focusing on Se compounds that exceeded the recommended threshold, particularly in combination with other cancer therapies [8]. The rationale behind this approach is based on in vivo evidence that demonstrates enhanced efficacy of chemotherapy drugs at higher doses, while protecting normal cells from the cytotoxic effects of anticancer therapy [8, 9]. Consequently, efforts to translate these promising preclinical models into clinical trials are essential to evaluate the potential of Se as a modulator of cytotoxic chemotherapy activity and toxicity. However, it is crucial to investigate thoroughly the safety and pharmacodynamic activity of various Se doses and compounds to optimise its combination with cancer therapies. In this pursuit, researchers from Waikato Hospital [10, 11] conducted a phase I randomised double-blinded trial involving 24 patients with chronic lymphocytic leukaemia (CLL) and metastatic solid malignancies. The patients were divided into three groups and randomised to take a daily oral dose of 400 µg Se over an 8-week period as sodium selenite (SS), SeMet, or Se-methylselenocysteine (MSC). Both SS and SeMet undergo metabolism to hydrogen selenide, a critical component for the synthesis of selenoproteins. Additionally, SeMet can be incorporated into proteins in place of methionine (Met) and is primarily stored in albumin [12, 13]. MSC, once absorbed, is transformed to methylselenol, which can either be demethylated to produce selenide for selenoprotein formation or methylated to generate volatile Se species for excretion, such as dimethylselenide or trimethyl selenonium ion [5]. The selection of these compounds was based on preclinical models indicating the superior short-term safety and effectiveness of organic compounds like SeMet and MSC compared to inorganic ones such as SS [8]. The study focused on assessing safety, tolerability, pharmacokinetic (PK) profiles, and pharmacodynamic (PD) parameters in plasma and peripheral blood mononuclear cells (PBMCs). At the administered dose, all three Se compounds were well-tolerated and found to be non-genotoxic [10, 11]. While the plasma Se level increased with the three Se compounds, no significant changes in PD parameters were observed at the dose of 400 µg/day. The investigators are now recruiting a further cohort of patients treated with higher doses to investigate these endpoints [11].

To the best of the authors’ knowledge, this trial represents the first attempt to compare three Se compounds administered orally within the same cancer population. However, information is still incomplete about the underlying mechanisms of Se as a chemopreventive reagent [14]. As Se and its species are considered good indicators of Se status in humans [13], their determination is crucial before initiating combined treatments involving Se and chemotherapy drugs. While Se metabolites like methylselenol have been suggested to be important mediating species in cancer prevention and treatment [5, 9, 13], the role of selenoproteins like selenoprotein P (SELENOP) has been increasingly acknowledged, not only in the context of cancer but also in other critical diseases such as diabetes and Alzheimer’s disease [1]. The main biomarkers in plasma/serum for assessing Se status include (a) SELENOP and glutathione peroxidase-3 (GPX3), which are the key and most abundant selenoproteins in this body fluid; (b) Se human serum albumin (Se-ALB), a Se-containing protein formed by the non-specific replacement of Met by SeMet in albumin; and (c) total Se, which contains SELENOP, GPX3, Se-ALB, plus low-molecular-weight (LMW) Se compounds that contribute < 3% of the total Se concentration [13, 15, 16]. Vicenti et al. [17] highlighted the importance and lack of data on the distribution and correlation of Se species with total Se in epidemiologic studies.

Interventional studies [5, 18] investigating different forms of Se at higher doses (≥ 400 µg/day) over short periods (up to 4 months) revealed that when supplementing Se-replete healthy individuals (mean baseline plasma Se concentration of 108 µg/L [5] and 122 ± 13 µg/L [18] with Se compounds (SS, SeMet, Se-yeast, and MSC), the levels of selenoproteins (SELENOP and GPX3) at the evaluated doses and compounds were not significantly affected. While the organic forms increased plasma Se levels in a dose-dependent manner (SeMet, Se-yeast > MSC), SS only had a significant effect at a higher dose (600 µg/day, 16 weeks). The increase of the plasma Se concentration after administration was, therefore, mainly attributed to the non-specific incorporation of Se into proteins (e.g. Se-ALB). Expression of SELENOP and GPX3 seemed to be optimised at the baseline plasma Se level, and the increase in urinary Se excretion was observed after treatment (SeMet > MSC or Se-yeast > SS), confirming that subjects were Se-replete [18]. On the other hand, Se biomarkers correlated with one another in Se-deficient [19, 20] or mild-to-moderate Se populations [21, 22].

For clinical purposes, selenoproteins, especially SELENOP, have usually been determined by ELISA [5, 23–25]. However, this immunoassay often faces challenges with selectivity, being isoform-specific, and yielding results associated with large measurement errors [5]. As an alternative approach, high-performance liquid chromatography coupled to inductively coupled mass spectrometry (HPLC-ICP-MS) has accurately estimated Se bound to SELENOP using reference methodologies based on isotope dilution analysis (IDA) [16, 26]. Despite their potential, studies based on the speciation of selenoproteins by HPLC-ICP-MS in interventional trials with Se compounds remain limited. This is probably due to the lack of validated and straightforward methods that can be easily applied to obtain multi-species information in numerous clinical samples of limited volumes.

From the foregoing account, the main goal of this study was to perform for the first time, a systematic study of the Se species distribution and characterisation in the plasma of cancer patients treated with increased Se levels induced by oral administration of a supranutritional dose (400 µg/day) of the Se compounds SS, SeMet, and MSC. This was undertaken as part of the aforementioned phase I randomised double-blinded trial [10, 11] by using methodology-based on HPLC-ICP-MS after sample preparation approaches and spiking experiments and, in comparison with total Se measurements, for mass balance purposes. Double-affinity HPLC-ICP-MS was conducted to determine simultaneously the main plasma/serum Se biomarkers (SELENOP, Se-ALB, GPX3, LMW Se species, and total Se in one analysis) in plasma samples collected before (baseline) and after 4 weeks of Se treatment. Total Se measurements and LMW speciation studies were also performed to obtain information on the distribution of Se species and their correlation with total Se levels. To gain a deeper understanding of the underlying mechanisms of SeMet and its effects, the incorporation of SeMet into proteins was explored in samples taken before and after Se administration, employing a combination of two different HPLC modes with ICP-MS and spiking experiments. The speciation data presented here will be invaluable in selecting the optimal form and dose to be applied in future clinical trials in combination with anti-cancer therapies.

## Experimental

### Reagents and standards

All reagents used were of at least analytical or high-purity grade. High purity deionised water (18.2 MΩ.cm) was obtained from an ELGA water purification system (Veolia Water, Marlow, UK). High purity nitric acid (HNO_3_), hydrogen peroxide (H_2_O_2_), hydrochloric acid (HCl), and single-element standard solutions with a concentration of 1000 mg L^−1^ of Se, germanium (Ge), and rhodium (Rh) for ICP-MS measurements were purchased from Romil (Cambridge, UK). An additional Se calibration standard (SRM 3149, primary standard) was obtained from a National Measurement Institute (NIST, Gaithersburg, USA). Calibration standards were prepared by diluting the stock standard with ultrapure water and nitric acid to the required concentrations and acid strength of 0.5% or 15% (v/v). HPLC mobile phases were prepared from ammonium acetate (NH_4_OAc), trifluoroacetic acid (TFA) obtained from Sigma-Aldrich (Gillingham, UK), optigrade methanol (MeOH) from LGC standards (Luckenwalde, Germany), and formic acid (FA) from Fisher Scientific (Loughborough, UK). The following small Se species standards were used for HPLC-ICP-MS: SS, sodium selenate, SeMet, MSC, seleno-L-cystine, methyl-2-acetamido-2deoxy1-seleno-β-D-galactopyranoside (selenosugar-1), methylselenic acid (MSA), and L-γ-glutamyl-Se-methylseleno-L-cysteine. The origin and preparation were previously reported [9, 27]. For enzymatic extractions, protease from *Streptomyces griseus*, lipase from *Candida rugosa*, and Trizma hydrochloride (Tris–HCl) and dithiothreitol (DTT) from Sigma-Aldrich were used. Amicon Ultra 0.5 mL centrifugal filters of 10 and 30 kDa cut-off were also obtained from Sigma-Aldrich.

The biological reference materials used for quality control (QC) purposes were the human serum CRM BCR-637 from the Institute for Reference Materials and Measurements (IRMM, Geel, Belgium) and the human plasma SRM 1950 Metabolites in Human Plasma from NIST.

### Study design and sampling

**Fig. 1:**
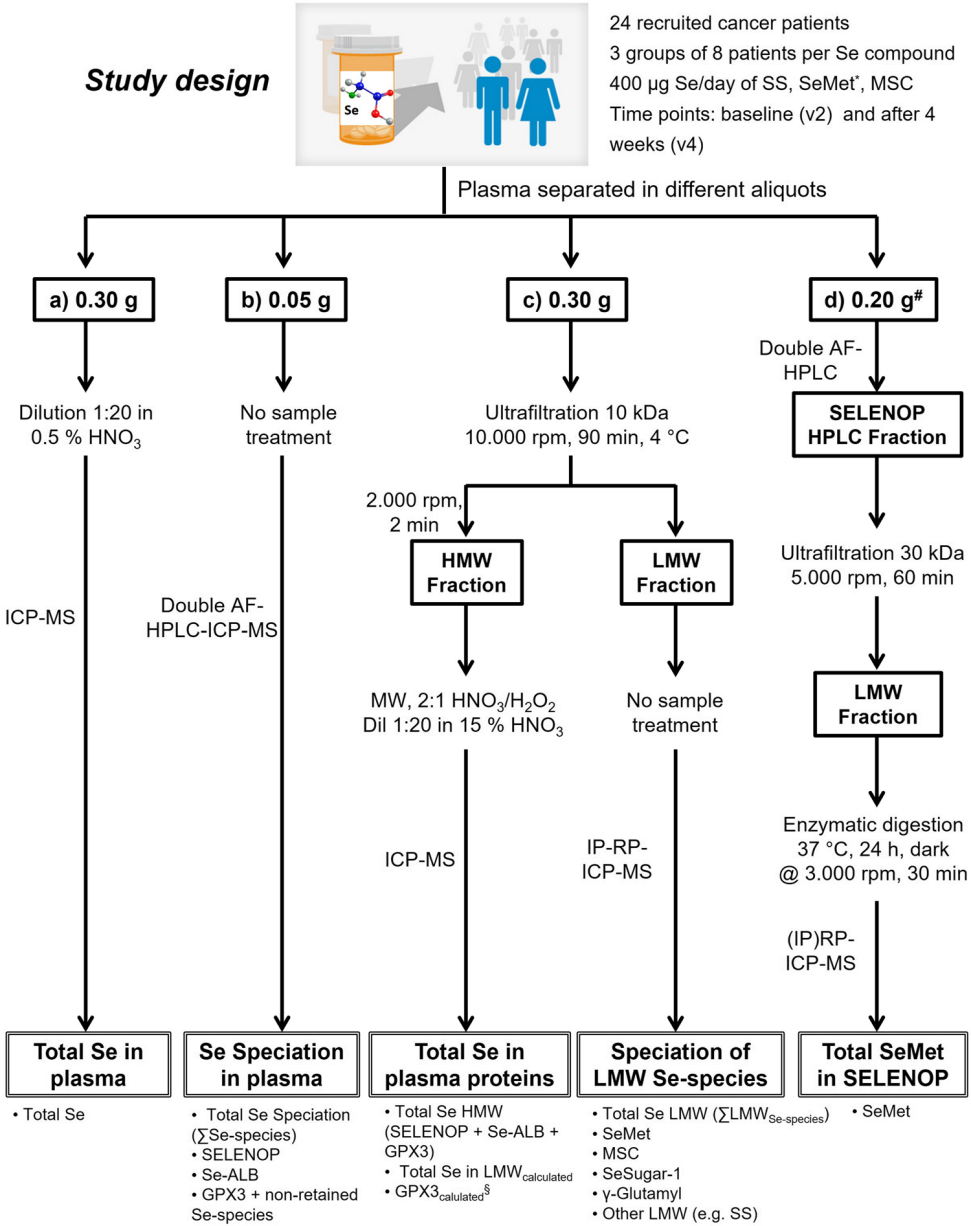
Bioanalytical workflow conducted for the assessment of plasma biomarkers of Se status (e.g. total Se, SELENOP, LMW Se species) in cancer patients from the phase Ib randomised double-blinded, multi-Se compound study of SS, SeMet, and MSC. *Only 7 patients; #only two pairs of SeMet administrated samples and CRM materials used

This work was part of the study registered under the Australian and New Zealand Clinical Trials Registry ACTRN12613000118707. A detailed description of the study and the baseline characteristics of the recruited patients have previously been published [10, 11]. Briefly, this phase Ib clinical trial was a randomised, double-blinded, dose-escalation study of three Se oral compounds: SS, SeMet, and MSC. Twenty-four participants with CLL or metastatic solid cancer, in whom the use of chemotherapy was not anticipated in the next 3 months, were randomised into 3 groups, each taking a single Se compound (SS, SeMet, or MSC) as one capsule daily, containing the equivalent of 400 μg/day of Se, for 8 weeks. Each cohort was balanced by disease type: 4 participants with CLL and 4 with solid cancers. Venous blood for Se measurements was taken at each study visit at Waikato Hospital (Hamilton, New Zealand) in trace element tubes (K2 EDTA, BD Biosciences, USA) and processed to produce plasma (spun at 1000 g for 10 min at 4 °C), which was stored at − 80 °C. Plasma samples (24 patients at two time points, approximately 3 mL volume), taken at baseline (on the same day the patients started the treatment but taken pre-dose) and on week 4, were received at LGC on dry ice and stored at − 80 °C until analysis. Samples from one participant who withdrew from the study were not analysed. A general density value of 1.025 kg L^−1^ was used to convert mass per mass results (µg kg^−1^) into mass per volume (µg L^−1^). A schematic representation of the bioanalytical workflow used to evaluate the responses of the different biomarkers of Se status for the three Se compounds (SS, SeMet, and MSC) in the patients’ samples before and after 4 weeks of treatment is shown in Fig. 1. A detailed description of the analytical procedures conducted in this study is given below.

### Instrumentation

A four-five decimal figure calibrated analytical balance (Cole-Parmer, UK) was used to prepare all solutions gravimetrically. A biosafety Class II cabinet (CAS BioMAT 2, Contained Air Solutions LTF, UK) was used when handling plasma samples. Microwave acid digestions were performed in 6 mL Teflon micro-inserts using the Ethos UP microwave system (Milestone S.r.l., Sorisole, Italy) prior to Se determination. Plasma samples were ultrafiltered by using a temperature-controlled centrifuge (5810R Eppendorf, Thermo Fisher Scientific, Germany). Enzymatic hydrolysis of plasma samples was performed in the dark using a hybridisation oven model Agilent G2545A (Sheldon Manufacturing, Inc., OR, USA).

HPLC measurements were carried out in a 1200 Series HPLC system (Agilent Technologies, Palo Alto, CA, USA) equipped with a binary pump, a thermostatted autosampler, and a 2-position 6-port switching valve. The separation of selenoproteins in plasma samples was done by double-affinity HPLC using HiTrap® Heparin and HiTrap® Blue High Performance affinity columns (1 mL, Sigma). The speciation of Se metabolites in LMW plasma ultrafiltered fractions was performed by Reversed-phase and Reversed-phase Ion-pairing HPLC (RP-IP-HPLC) using an Agilent Zorbax C_8_ column (250 × 4.6 mm i.d.; 5 µm). The HPLC system controlled by the ChemStation software (B.01.03-SR2, Agilent Technologies, USA) was connected to the ICP-MS via a PEEK tubing (0.1 mm i.d., Sigma). Elemental Se and speciation analysis were performed using a collision/reaction cell Agilent 8800 ICP-MS/MS instrument (Agilent Technologies) controlled by the MassHunter software (v.4.5, Agilent). The instrument was equipped with a MicroMist nebuliser, a Peltier-cooled (2 °C) Scott spray chamber, and nickel cones. The ICP-MS was operated in tandem mass spectrometry (MS/MS) mode using oxygen (O_2_) and hydrogen (H_2_) gases with a mass shift of *m/z* + 16 to reduce and overcome interferences on the selected isotopes ^77^Se, ^78^Se, and ^80^Se. The instrument was tuned daily for maximum sensitivity on the selected Se isotopes using a 10 µg kg^−1^ Se standard in either 2% (v/v) HNO_3_ or the corresponding mobile phase. Operational conditions and data acquisition parameters are given in Table S1 (ESI).

### Analytical procedures

#### Fractionation of Se species in plasma samples

Approximately 0.30 g of plasma was accurately weighed in pre-cleaned Amicon Ultra 0.5 mL 10 kDa cut-off centrifugal filter devices (Fig. 1c). Samples were spun at 10.000 rpm for 90 min at 4 °C to separate the HMW protein fraction (retentate or supernatant) from the LMW fraction (filtrate). Procedural blanks and BRC-637 untreated and spiked with SeMet before and after the ultrafiltration process were run in parallel for QC purposes. The HMW fraction was then recovered from the filter by spinning the filter device at 2.000 rpm for 2 min. Both fractions were subsequently reconstituted to the original volume (0.30 g) in physiological media (e.g. 50 mM Tris–HCl, pH 7.4). The HMW fraction was then submitted to microwave digestion and ICP-MS analysis for the determination of total Se in plasma proteins. Speciation of small Se species was performed in the LMW fraction by IP-RP-ICP-MS analysis.

#### Determination of total Se in plasma samples and HMW fractions by ICP-MS

The analysis of total Se in plasma samples was conducted by the sample dilution approach [27]. Briefly, approximately 0.30 g of each plasma sample was accurately weighted into a 15 mL tube containing 0.5% (v/v) of HNO_3_ (Fig. 1a). An internal standard (IS) mixed solution of approximately 40 µg kg^−1^ Ge and 10 µg kg^−1^ Rh, in 0.5% HNO_3_ and 2% MeOH (v/v), was used online to correct for any instrumental drift and ionisation effects. Samples prepared in duplicate were directly submitted to ICP-MS analysis with a total dilution factor of approximately 20. On the other hand, the determination of total Se in the ultrafiltered HMW fractions (Se species > 10 kDa, Fig. 1c) was performed by MW-assisted acid digestion in closed vessels. The reconstituted HMW samples (~ 0.30 g) were accurately weighted into 6 mL Teflon micro-inserts using a mixture of HNO_3_ and H_2_O_2_ (2:1 v/v). The following acid digestion program was applied: 0 to 180 °C in 10 min (1000 W) and held at 180 °C for 10 min (1000 W). The digests were made up to a final volume of ~ 5 g with deionised water containing Ge and Rh as IS and 2% (v/v) MeOH and directly submitted to ICP-MS analysis without further dilution. Additionally, reagent blanks, two samples of BCR-637 certified for their total Se concentration (79 ± 7 µg kg^−1^ Se, *k* = 2) [16], and spiked samples with inorganic Se were also submitted to the same procedures for accuracy assessment. Total Se determination was performed by external calibration using matrix-matched Se calibration standards. Working standards and an independent Se solution were prepared daily by gravimetric dilution in either 0.5% HNO_3_ for total Se analysis in plasma or 15% HNO_3_ in the HMW protein fractions to match the acid concentration in the samples. The working range for the standards was 0 to 40 µg kg^−1^ Se. For the calculation of the limit of detection (LOD) and quantification (LOQ), at least 5 blanks were run in each analytical batch. Data was processed and results calculated using the MassHunter software. For all the analyses presented in this work, calibration curves achieved correlation coefficients of at least 0.995, and results are reported for the isotope ^78^Se and expressed as µg kg^−1^ Se. Acquisition parameters are summarised in Table S1 (ESI).

#### Analysis of Se species in plasma by double-affinity HPLC-ICP-MS

The separation of Se species in the plasma samples was carried out by the combination of two affinity columns prepacked with heparin and blue sepharose media, with the first column retaining SELENOP and the latter albumin or Se-ALB (Fig. 1b) [16]. Se species not retained with this setup (eluting in the void peak) include GPX3 and LMW Se species such as SeMet and inorganic Se. Elution of the Se species in the plasma samples was achieved by a combination of ammonium acetate buffers at different molar strengths at a flow rate of 0.5 mL min^−1^ using a gradient in valve-switching mode (Table S1, ESI). The quantification of the Se mass fraction bound to each chromatographic peak was performed by online external calibration using inorganic Se standards. A Se calibration standard curve was prepared gravimetrically in ultrapure water containing 0 to 200 µg kg^−1^ Se and an independent Se solution in the mid-calibration range was used for QC. To mimic the double AF-HPLC separation conditions (matrix-matching), Se inorganic standards were eluted with the binding buffer for the quantification of the first or void peak and with the eluting buffer for SELENOP and Se-ALB. The Se levels associated with SELENOP and Se-ALB were determined directly from the Se signals in the double-affinity chromatograms. However, since GPX3 elutes with other non-retained Se species (e.g. SeMet), an indirect approach was used to determine its Se content. This was done by subtracting the sum of Se associated to SELENOP and Se-ALB from the total Se in the HMW fraction (SELENOP + Se-ALB + GPX3).

Plasma samples without any treatment were injected in duplicate and run in 4 different batches. Each batch contained at least 5 blanks to calculate LOD/LOQ and the NIST SRM 1950 as QC material injected along the sequence, bracketing the samples, to calculate repeatability, specificity, and recovery, the main components of the uncertainty budget. The integration of the chromatographic peaks was manually performed using the MassHunter software and data transferred to Microsoft Excel 2016 (Microsoft, WA, USA) for further treatment. The sum of the 3 chromatographic peaks was expressed as the sum of Se species (∑Se species) and compared to the total Se value obtained by ICP-MS.

#### Speciation of LMW Se species by IP-RP-HPLC-ICP-MS

The LMW fractions obtained after the ultrafiltration of plasma patient samples using the 10 kDa cut-off membranes filters were analysed by IP-RP-ICP-MS without further treatment (Fig. 1c) previously described elsewhere [27, 28]. IP-RP-HPLC separation was performed with an isocratic flow of an aqueous mobile phase containing 2% (v/v) MeOH and 0.1% (v/v) TFA (ion-pairing reagent) and the addition of ~ 20 µg kg^−1^ Rh in 0.5% HNO_3_ and 4% MeOH (v/v) as IS to correct for any instrumental drift (Table S1, ESI). Quantification was performed via external calibration using SeMet standards. The SeMet stock standard was of high purity and fully characterised in-house in terms of its Se content by IDA (400.4 ± 7.8 µg kg^−1^, *k* = 2). Calibration standards in the range of 0 to 25 µg kg^−1^ as Se were prepared by diluting the stock solution in water containing 0.02% DTT to avoid the oxidation of SeMet. Following the previously described *double AF-HPLC procedure*, LMW plasma fractions were also analysed in 4 batches. Each batch was composed of samples injected twice, blanks, an independent commercial SeMet standard (Sigma), and two LMW BCR-637 fractions spiked before and after with SeMet for QC purposes. Data treatment was conducted as described above. The identity of Se species was confirmed in plasma patient samples by spiking experiments with corresponding selenosugar-1, SeMet, and MSC species.

#### Characterisation of SeMet in the SELENOP affinity fraction

The following procedure was performed to obtain further evidence about the non-specific incorporation of SeMet into SELENOP after SeMet administration. Two complete pairs of plasma samples from patients treated with SeMet (Pt2&24, unblinded after the end of the study), blanks, and the SRM 1950 were analysed by double AF-HPLC (Fig. 1d). Corresponding HPLC fractions of SELENOP (13–16 min) from two independent injections of the patients’ samples were collected and combined. HPLC fractions were pre-concentrated using 30 kDa filters (5.000 rpm, 30 min). The HMW fraction was transferred to a pre-cleaned 2 mL snap-cap vial together with 2-time washes of the filter with 100 µL of the affinity elution buffer. Then, approximately 250 µL of the digestion buffer (60 mM Tris–HCl in 0.02% DTT, containing 0.40 g of protease and 0.20 g of lipase per 10 mL buffer) was added and the enzymatic digestion was performed at 37 °C for 24 h in the dark with rotation. After digestion, samples were centrifuged at 3.000 rpm for 30 min and stored at − 80 ºC until analysis. Digests were analysed by IP-RP-ICP-MS/MS for the determination of SeMet. Complementary RP-ICP-MS/MS using 0.2% (v/v) FA was additionally performed and samples spiked with SeMet were also analysed for the identification of SeMet.

### Statistical analysis

Statistical tests were performed in Excel with the Data Analysis ToolPak (Microsoft Office Professional Plus 2016). Paired two-tailed *t*-tests (paired two sample for means) with a 95% confidence level were applied to compare biomarkers of Se status before and after administration. The results were considered statistically significant when the *p* value was less than 0.05, and differences were marked as follows: *p* < 0.05 (*), *p* < 0.01 (**), and *p* < 0.001 (***). The relationship between parameters was tested by Pearson’s correlation analysis, considering the following criteria for the correlation coefficient (*r*): very high correlation (0.9 to 1.0), high correlation (0.7 to 0.9), moderate correlation (0.5 to 0.7), and low or negligible correlation (≤ 0.5).

## Results and discussion

The study design and the analytical workflow are illustrated in Fig. 1, and the results of these analyses are discussed hereunder.

### Assessment of Se levels in the different plasma compartments before and after administration

The total Se content in whole plasma was determined as a primary biomarker of Se status (Fig. 1a). To achieve this, a simple and straightforward approach was conducted by diluting the plasma in 0.5% (v/v) HNO_3_ to avoid protein precipitation [27]. The method was validated, and a complete uncertainty budget was estimated (expanded uncertainty, *U*_c_ = 10%, *k* = 2) considering repeatability (3.6%), specificity (0.3% ^78^Se vs ^80^Se), and accuracy (96.7% recovery from analysis of BCR-637 and spiking experiments). The method showed a good sensitivity with LODs and LOQs of about 2.2 and 6.4 µg kg^−1^ for ^78^Se, respectively, for an average 20-fold dilution. Table 1 summarises total Se plasma levels and individual data for each randomised Se compound group at the two time points of the study. Baseline mean plasma Se levels of 93.7 ± 8.7 µg kg^−1^ Se (IQR; 83.9–103.1 µg kg^−1^ Se) were observed in this study in agreement with data previously reported (10). These values in New Zealand participants are also within the range reported for healthy Europeans [27, 29, 30]. Plasma Se concentrations increased significantly after 4 weeks of Se administration of the 3 compounds (*p* < 0.001) and half of the subjects showed a Se rise higher than 50%. Moreover, subjects with the highest baseline Se levels (≥ 107 µg kg^−1^ Se) showed the lowest Se increase (≤ 12%). Total Se levels increased most after the administration with SeMet (134% ± 22%), while a moderate increase (~ 30%) was observed for SS and MSC treatment, as previously found in other Se trials [5, 18, 23, 27].

**Table 1:** Total plasma Se concentrations by ICP-MS (Fig. 1a) at baseline (v2) and after 4 weeks of administration (v4) expressed as µg kg^−1^ Se: mean ± expanded uncertainty (2 independent measurements per sample, *k* = 2), median, interquartile range (IQR) in all study subjects (*n* = 23) and in the randomised groups (SS, SeMet, and MSC). Results of the comparison of the two time points expressed as increase of Se (ΔSe, %) and *p*-value (paired *t*-test): *p* < 0.001 (***)

	Total plasma Se				
Sample	Mean	Median	IQR	ΔSe, %	*p*-value
v2, *n* = 23	93.7 ± 8.7	89.6	83.9–103.1	62 ± 12	3.5 × 10^−5^ (***)
v4, *n* = 23	147.7 ± 21.7	126.4	115.5–174.0		
SS v2, *n* = 8	94.1 ± 13.1	91.1	84.3–101.3	27 ± 7	4.0 × 10^−3^ (***)
SS v4, *n* = 8	116.7 ± 8.5	115.4	112.7–121.2		
SeMet v2, *n* = 7	90.9 ± 13.9	89.6	85.4–92.1	134 ± 22	4.1 × 10^−4^ (***)
SeMet v4, *n* = 7	208.1 ± 31.6	190.4	184.8–225.2		
MSC v2, *n* = 8	95.7 ± 12.6	87.5	83.0–107.2	33 ± 6	8.6 × 10^−4^ (***)
MSC v4, *n* = 8	125.9 ± 15.8	119.4	117.2–131.8		

In parallel, total plasma Se concentrations were also obtained by the double AF-HPLC speciation approach (Fig. 1b) considering the sum of all Se species (∑Se species). This methodology, validated using the SRM 1950 certified in total Se, provided an average recovery of around 94% (99.4 ± 17.8 µg kg^−1^ Se, *U*_c_ = 18%, 4 independent batches). High positive correlations (*r* > 0.9) with total Se by ICP-MS were obtained for all randomised groups (Table S2, ESI) pointing out the feasibility of this speciation strategy to provide reliable total Se levels using limited sample volume and analysis time (50 µL, 35 min).

The determination of total Se in the HMW fraction or selenoproteins’ pool (Fig. 1c) was conducted by microwave-assisted digestion of the ultrafiltered plasma fraction (> 10 kDa), ensuring a complete digestion of the proteins [27]. Quantitative recoveries from the ultrafiltration process were obtained in terms of mass (98.8% ± 1.5%, *U*_c_ = 3%) and Se content (104.1 ± 2.9 µg kg^−1^ Se, *U*_c_ = 6%, 2 independent batches, BCR-637). Moreover, no levels of Se above LOQ were observed from the centrifugal filter devices making it suitable for the fractionation of Se species in plasma. Total Se levels in the HMW fraction (Table S3, ESI) before and after treatment were ≥ 95% of total Se and showed the same behaviour observed previously for total Se plasma. In this case, moderate to high correlations (0.5 > *r* > 0.9) with total Se plasma were obtained, and all treated groups showed high correlations (*r* > 0.8) except for MSC (*r* = 0.5), indicating a possible higher Se excretion in this group. Consequently, total Se in the LMW pool, calculated indirectly by the subtraction of the Se content in the HMW pool from total plasma Se, represented ≤ 5% of the total plasma Se, and no significant changes were observed among the Se compound groups, as previously reported in the literature [27, 30, 31]. Therefore, speciation studies of both LMW and HMW Se pools are of utmost importance to elucidate the impact of each Se compound on the Se distribution in plasma.

### Distribution of plasma LMW Se metabolites in response to SS, SeMet, and MSC administration of cancer patients

The determination of LMW Se species was conducted in the plasma filtrates (< 10 kDa or LMW Se pool) by using an established IP-RP-ICP-MS methodology that is able to resolve the most common Se metabolites found in biological samples (e.g. SeMet, MSC, and selenosugar-1) [27]. Inorganic Se species (SS and sodium selenate), not retained using this setup, elute in the void volume together with salts and other non-retained Se species such as MSA and seleno-L-cystine. Quantification of Se bound to each LMW Se species was performed using an *in-house* characterised SeMet standard taking advantage of the species-independent ionisation properties of the ICP-MS and the isocratic chromatographic condition. The accuracy of the quantification method over 4 independent batches determined by an independent SeMet standard was (102.2% ± 4.6%) and precisions lower than 5% in the plasma matrix were obtained. Moreover, human serum BCR-637, in which SeMet concentrations were found < LOQ, was spiked with SeMet before and after being submitted to the ultrafiltration procedure. The average recovery from the analysis of the BCR-637 sample spiked with SeMet was (102.2% ± 2.2%) (*n* = 4), verifying the suitability of the fractionation procedure. Average LODs and LOQs were 0.15 and 0.45 µg kg^−1^ Se for SeMet, 0.20 and 0.60 µg kg^−1^ Se for selenosugar-1, and 0.11 and 0.32 µg kg^−1^ Se for MSC, respectively. Only selenosugar-1, MSC, and non-retained species showed concentrations above their corresponding LOQs.

**Fig. 2:**
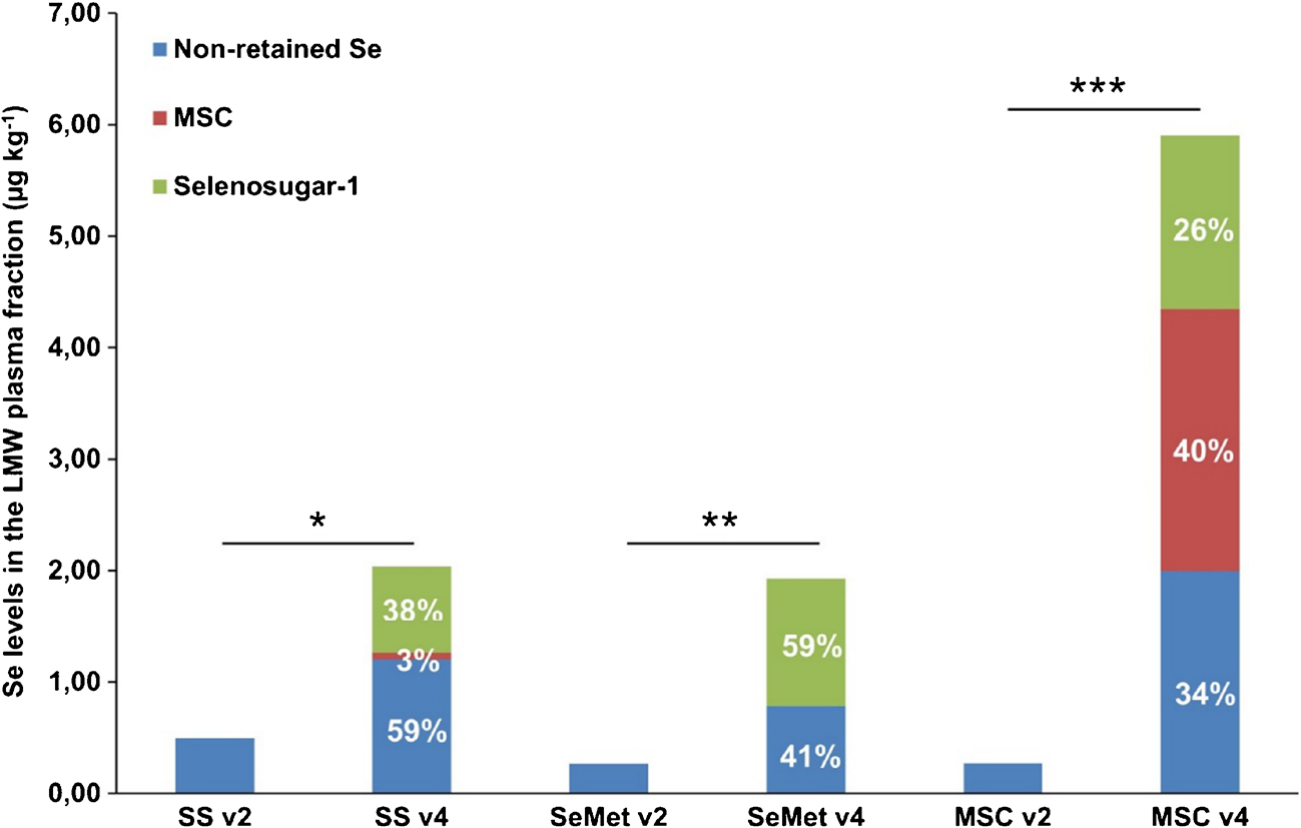
Level of LMW Se species detected (non-retained Se, MSC, and selenosugar-1, expressed as µg kg ^−1^ Se) in the plasma filtrates (< 10 kDa) of patients randomised in the three Se compound groups (SS, SeMet, and MSC) before (v2) and after treatment (v4) by IP-RP-ICP-MS. Distribution of Se species in the administered groups expressed as percentage. Analysis of selenosugar-1 levels before and after supplementation done by paired two-tailed *t*-test; *p* < 0.05 (*), *p* < 0.01 (**), and *p* < 0.001 (***)

Figure 2 shows the levels of Se found in the LMW plasma fractions from the three randomised groups before and after treatment and the distribution of LMW Se species after treatment. At baseline, only non-retained Se species were quantified (≤ 0.5 µg kg^−1^ Se). While the LMW Se plasma pool increased after administration of each Se compound, the increase was greater after MSC (≤ 6.0 µg kg^−1^ Se, ~ 4.5% of total Se plasma) than after either SS or SeMet (≤ 1.9 µg kg^−1^ Se). The composition of the LMW fraction is shown in Fig. 2; selonosugar-1 accounted for 26–59%, and non-retained Se species for 34–59% of the total Se in the LMW fraction. On the other hand, MSC was only detected after administration of SS (3%) or MSC (40%). Selenosugar-1 was found to be the most abundant LMW plasma metabolite overall, particularly with MSC (*p* = 1.3 × 10^−4^), consistent with previous studies of Se supplementation [27, 30]. The moderate correlation (*r* = 0.5) found previously between total Se and the HMW Se pool with MSC is consistent with more MSC being excreted via the metabolites in the LMW fraction.

Figure S1 (ESI) shows representative chromatograms of selected patient samples (Pt12, Pt11, and Pt8) both before and after treatment with the three Se compounds. Selenosugar-1 (Fig. S1a), SeMet (Fig. S1b), and MSC (Fig. S1c) were identified, respectively, in the plasma patient samples by spiking experiments, confirming the identity of these Se metabolites.

### Assessment of changes in the HMW Se plasma pool after Se administration

Since the impact of Se treatment in the response of the LMW Se plasma pool was minimal (≤ 5% Se of total plasma Se), special attention was further paid to investigate changes in the HMW Se plasma pool of patients administered with the three Se compounds. A fit-for-purpose, multi-sample approach for Se speciation in plasma, easily implemented for clinical measurements, was developed and validated against IDA values [16, 26]. The double AF-HPLC-ICP-MS speciation methodology (Fig. 1b) allowed simultaneous quantification of Se associated with SELENOP, Se-ALB, and GPX3, along with non-retained species, and the sum of Se species (∑Se species), as previously reported [16]. As an example, Figure S2 (ESI) depicts double AF-HPLC-ICP-MS Se chromatograms of a patient (Pt2) before and after SeMet administration. The method was validated by using the NIST SRM 1950 (Table S4, ESI) and a complete uncertainty budget was estimated with recoveries between 92.0 and 103.3%. The expected values of the SRM 1950 correspond to the more accurate values obtained so far, using IDA. The Se value for SELENOP (60.6 ± 3.2 µg kg^−1^, *k* = 2) was obtained by double species-specific IDA in combination with HPLC-ICP-MS/MS at the peptide level [26], while the total Se (105.5 ± 3.8 µg kg^−1^, *k* = 2.2) in the SRM 1950 is the reference value determined by IDA-ICP-MS by NIST. On the other hand, Se values for the first peak (GPX3 and non-retained Se species: 15.1 ± 1.7 µg kg^−1^, *k* = 2) and Se-ALB (23.1 ± 3.7 µg kg^−1^, *k* = 2) were obtained by post-column IDA-ICP-MS [16] due to the lack of species-specific protein standards.

#### A. SELENOP levels in cancer patients and correlation with total Se

As can be seen in Table 2, the subjects enrolled in this study exhibited at baseline, median Se bound to SELENOP levels of 63.3 µg kg^−1^ Se (IQR; 58.8–71.4 µg kg^−1^ Se) or 4.2 mg L^−1^ (IQR; 3.9–4.7 mg L^−1^), expressed as protein [25, 32]. SELENOP concentration (as mg L^−1^) was calculated following the equation described elsewhere [25], a plasma density of 1.028 kg L^−1^, an average molecular weight of SELENOP of 51,000 g moL^−1^ and of Se of 78.96 g moL^−1^, and an average Se content of SELENOP of 10 atoms of Se per SELENOP (considering 10 selenocysteines residues in human SELENOP) [13]. In this sense, SELENOP accounts for approximately 65–70% of the total Se plasma and closely aligns with the mean European value from the EPIC study (4.4 mg L^−1^ SELENOP) [29]. SELENOP levels increased with administration of all three Se compounds (Table 2), in a pattern similar to the increase in total plasma Se levels (Table 1); SeMet administration led to a notable increase in Se bound to SELENOP ((85% ± 17%) or 3.6 mg L^−1^ SELENOP) than administration of SS or MSC (~ 30% or 1.3 mg L^−1^ SELENOP). This resulted in a higher plasma SELENOP concentration after 4 weeks of treatment with SeMet (median 8.0 mg L^−1^) than with MSC or SS (median 5.5 mg L^−1^). These values agree with previous supplementation studies [19, 22, 23], reporting maximum SELENOP concentrations in the range of 5 to 7 mg L^−1^ (plateau concentration). In the case of SeMet administration, 5 out of 7 patients exhibited concentrations exceeding 7 mg L^−1^ SELENOP, in line with Brodin et al*.* [24] who observed higher plasma SELENOP levels beyond the plateau under high therapeutic Se supply (i.e. SS at high doses, > 1000 µg per day). In contrast, oral administration of SeMet or MSC at 400 µg or 800 µg daily for 84 days did not show a consistent increase in plasma SELENOP, nor evidence of a dose–response, albeit in subjects with a mean baseline plasma SELENOP of 7.4 mg L^−1^, which is considered to be in the Se-replete range [5]. As shown in Table 2, the correlation between total Se and SELENOP levels, at baseline and after administration with the three Se compounds, was very high (*r* ~ 0.9, *p* < 0.001). This suggests that subjects were Se-deficient before administration and that SELENOP was not fully expressed in most patients after treatment with any of the three compounds [19–22]. These results, and those of the trials referenced above, indicate that the dose response of different Se compounds for saturation of SELENOP plasma is not yet determined, but may be further elucidated with the higher doses of the three Se compounds (1600 µg and 6400 µg of Se day for 28 days) planned for this trial in New Zealand patients [11].

**Table 2:** Concentrations of Se bound to SELENOP obtained by double AF-ICP-MS at baseline (v2) and after 4 weeks of administration (v4) expressed as µg kg^−1^ Se: mean ± expanded uncertainty (2 independent measurements per sample, *k* = 2), median, interquartile range (IQR) in all study subjects (*n* = 23) and in the randomised groups (SS, SeMet, and MSC). SELENOP concentration expressed as mg L^−1^ quoted in brackets^a^. Results of the comparison of the two time points expressed as increase of Se (ΔSe, %) and *p*-value (paired *t*-test). And results of Pearson’s correlation analysis (correlation coefficient, *r*) between total Se by ICP-MS (Table 1) and double AF-ICP-MS. *p* < 0.01 (**), and *p* < 0.001 (***)

	Se bound to SELENOP					
Sample	Mean, Se/µg kg^−1^ (SELENOP/ mg L^−1^)	Median, Se/µg kg^−1^ (SELENOP/mg L^−1^)	IQR, Se/µg kg^−1^ (SELENOP/mg L^−1^)	ΔSe, %	*p*-value	*r*
v2, *n* = 23	65.3 ± 5.5 (4.3 ± 0.4)	63.3 (4.2)	58.8–71.4 (3.9–4.7)	29 ± 5	2.3 × 10^−6^ (***)	0.9
v4, *n* = 23	94.6 ± 10.2 (6.3 ± 0.7)	84.4 (5.6)	81.6–100.0 (5.4–6.6)			0.9
SS v2, *n* = 8	65.9 ± 9.3 (4.4 ± 0.6)	64.3 (4.3)	58.8–73.0 (3.9–4.8)	28 ± 7	2.6 × 10^−3^ (**)	0.9
SS v4, *n* = 8	83.1 ± 8.7 (5.5 ± 0.6)	83.4 (5.5)	76.3–89.4 (5.1–5.9)			0.9
SeMet v2, *n* = 7	66.1 ± 11.5 (4.4 ± 0.8)	66.3 (4.4)	60.1–72.7 (4.0–4.8)	85 ± 17	1.2 × 10^−3^ (**)	0.9
SeMet v4, *n* = 7	119.2 ± 19.0 (7.9 ± 1.3)	120.1 (8.0)	105.9–134.5 (7.0–8.9)			0.9
MSC v2, *n* = 8	64.1 ± 7.7 (4.3 ± 0.5)	62.8 (4.2)	60.3–63.9 (4.0–4.2)	34 ± 6	5.8 × 10^−4^ (***)	0.9
MSC v4, *n* = 8	84.4 ± 8.3 (5.6 ± 0.6)	82.4 (5.5)	80.8–85.0 (5.4–5.6)			0.9

^a^The SELONOP concentration (as mg L^−1^) was calculated following the equation described elsewhere (25), a plasma density of 1.028 kg L^−1^, an average molecular weight of SELENOP of 51,000 g moL^−1^ and of Se of 78.96 g moL^−1^, and an average Se content of SELENOP of 10 atoms of Se per SELENOP (considering 10 selenocysteines residues in SELENOP from human) (13)

#### B. Selenoalbumin increased significantly with SeMet administration

**Fig. 3:**
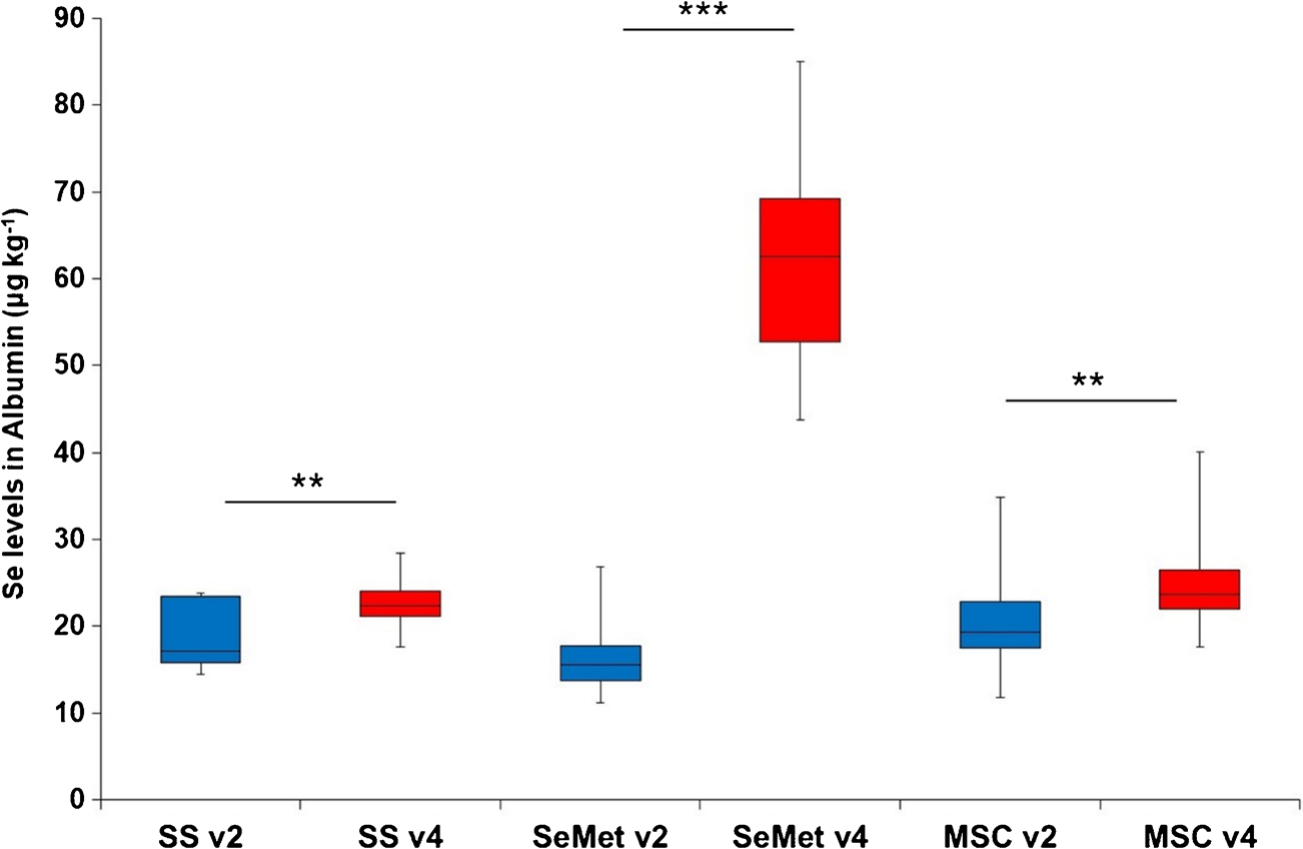
Boxplots representing the levels of Se bound to albumin (expressed as µg kg^−1^ Se, 2 independent measurements per sample) in the plasma of cancer patients (*n* = 23) before (v2) and after administration (v4) with SS, SeMet, and MSC by double AF-HPLC-ICP-MS. Statistical analysis by paired two-tailed *t*-test; *p* < 0.01 (**), and *p* < 0.001 (***)

Figure 3 presents the levels of Se bound to albumin (Se-ALB) represented as boxplots in the three randomised groups before and after administration with SeMet. The baseline median level of Se-ALB was 18.8 µg kg^−1^ Se (IQR; 15.5–23.0 µg kg^−1^ Se) accounting for approximately 20% of the total Se in the plasma. After administration, the Se concentration increased significantly by nearly 1.5 times to 25.4 µg kg^−1^ Se for the whole trail cohort, and with each of the three Se compounds (Table S5, ESI). As previously observed for total Se and SELENOP concentrations (Table 1 and 2, respectively), Se-ALB concentration showed a greater increase with SeMet (288%, *p* < 0.001), but a lesser increase with SS and MSC (~ 25%, *p* < 0.01). This outcome was expected, as albumin is the most abundant plasma protein and has six Met positions available for the non-specific incorporation of SeMet in lieu of Met [5, 13, 19]. Additionally, high positive correlation values between total Se and Se-ALB (*r* ≥ 0.8) were obtained after treatment [19, 23], while a moderate correlation was found for MSC (*r* = 0.5), consistent with previous observations for total Se in the HMW pool (Table S3, ESI), suggesting a potentially higher elimination of Se in plasma after oral MSC administration.

#### C. Glutathione-peroxidase 3 (GPX3) and distribution of Se species after treatment

Participants in this trial were found to contain baseline mean concentrations of GPX3 [calculated as total Se in the HMW_pool_ – (SELENOP + Se-ALB)] of 8.8 ± 0.9 µg kg^−1^ Se (IQR; 2.1–13.7 µg kg^−1^ Se) (Table S6, ESI). This represents less than 10% of the total Se in the plasma. After treatment with the Se compounds, values nearly doubled to 18.7 ± 2.2 µg kg^−1^ Se (IQR; 4.4–24.3 µg kg^−1^ Se), indicating Se-deficient subjects at baseline. Despite observing changes in GPX3 levels, they were not statistically significant for any of the three administered compounds. This study was unable to measure directly GPX3 activity in the plasma; this is unfortunate as this could provide more insights into the effect of Se compounds on the expression of this selenoprotein. Further investigations using direct GPX3 measurements and standardised protocols might help in comparing GPX3 values reported as glutathione peroxidase activity (units L^−1^) [5, 18, 19, 23] or expressed as Se (µg L^−1^) [16, 17].

**Fig. 4:**
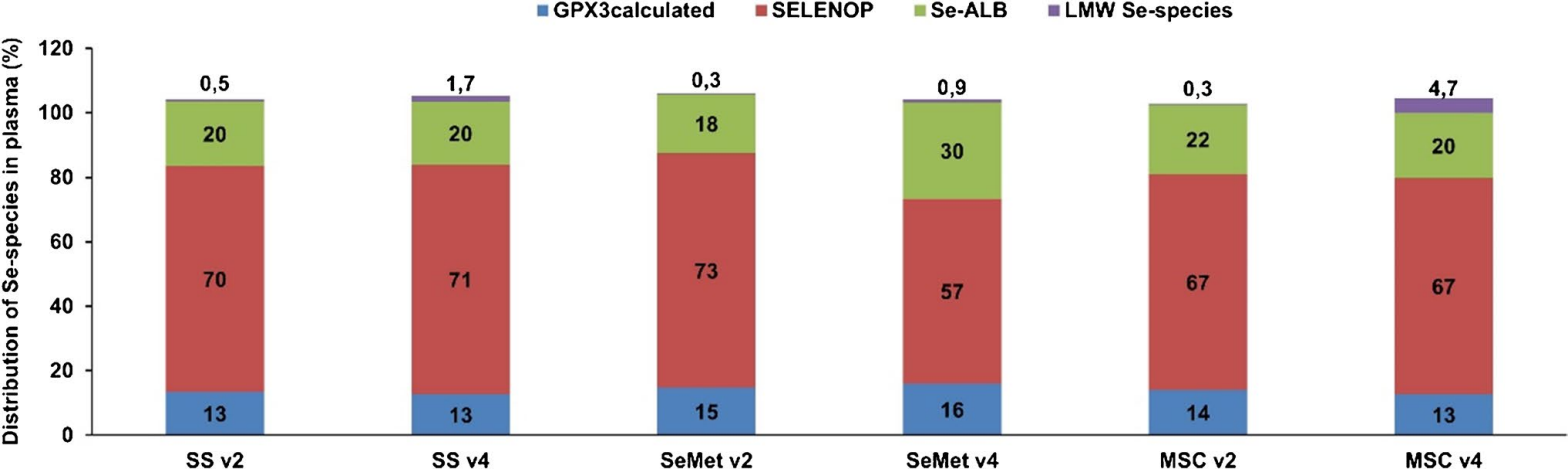
Distribution of Se species (GPX3_calculated_, SELENOP, Se-ALB, and LMW Se species), expressed as percentage vs total Se, in the plasma patient samples (*n* = 23) randomised in the three Se compound groups (SS, SeMet, and MSC) before (v2) and after (v4) administration

Figure 4 illustrates the distribution of Se species (GPX3_calculated_, SELENOP, Se-ALB, LMW_pool_) in comparison to total Se plasma for the three Se compounds, both before and after treatment. At baseline plasma levels (median total Se of 89.5 µg kg^−1^ total Se), SELENOP corresponded with the largest Se fraction (~ 70%), followed by Se-ALB (~ 20%), GPX3 (~ 10%), and the LMW_pool_ (≤ 0.5%) [16, 23]. Se administration positively affected all Se plasma biomarker levels, confirming Se deficiency in individuals. Small changes in the distribution of Se species were observed for SS and MSC, with a slight impact on the LMW_pool_ from MSC. However, SeMet administration led to significant changes in the Se species distribution; the percentage of Se-ALB (versus total Se) increased from 18 to 30%. These findings suggest that the albumin pool was the major contributor to the overall plasma Se distribution changes when subjects were administered SeMet.

#### D. Study of the incorporation of SeMet into the SELENOP affinity fraction

**Fig. 5:**
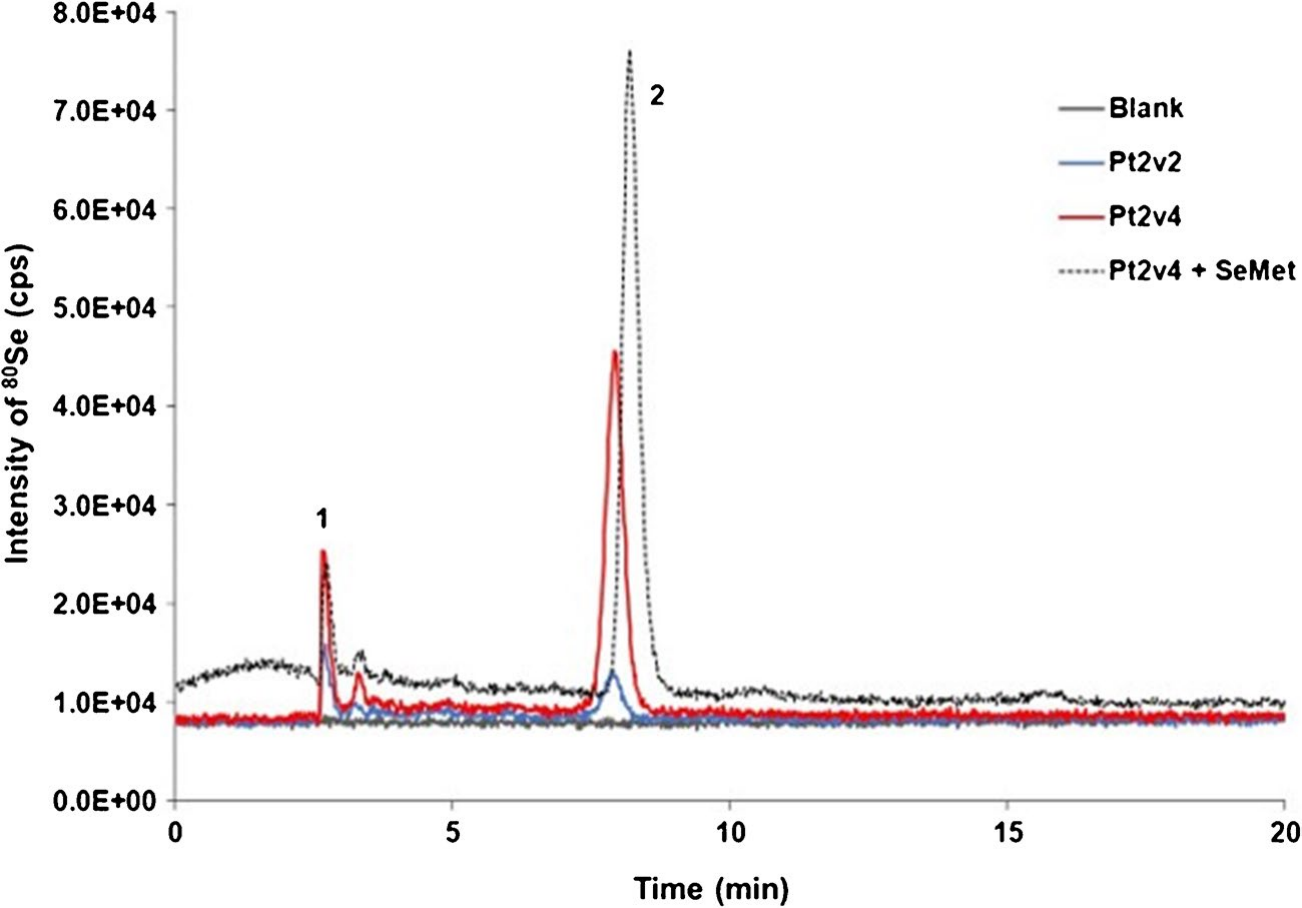
IP-RP-ICP-MS chromatograms of the enzymatic digestion of SELENOP affinity plasma fractions from Patient 2 (Pt2, administered with SeMet). Grey line: procedural blank, blue line: baseline sample (Pt2v2), red line: sample after treatment (Pt2v4), and dotted line: spiked with SeMet. Peak identification: (1) non-retained Se, (2) SeMet

Since SeMet administration produced a remarkable increase in absolute SELENOP levels, further experiments were conducted to elucidate the nature of this finding. Previous studies have reported two different Se metabolic routes for SeMet in humans [12, 15, 18]: (a) it can enter the non-regulated pool via non-specific incorporation into proteins at Met positions, and be stored mainly in albumin, but potentially in all proteins that contain Met; or (b) it can be incorporated into SeCys-containing proteins (e.g. SELENOP) via hydrogen selenide. To the authors’ knowledge, apart from the cell-free protein *E. coli* synthesis of a full-length human recombinant SeMet-SELENOP standard [33], the in vivo incorporation of SeMet into SELENOP in a real sample has not been demonstrated so far. SeMet and canonical Met exhibit similar efficiency in their incorporation into the protein structure. Furthermore, SELENOP contains four possible canonical Met. In this sense, plasma samples from two patients (Pt2 and Pt24) before and after SeMet administration were re-analysed by double AF-ICP-MS (Fig. 1d). The corresponding SELENOP affinity fractions were collected, pre-concentrated, and enzymatically digested for the potential release of endogenous SeMet. Figure 5 (and Figure S3a, ESI) shows the IP-RP-ICP-MS chromatograms (using 0.1% TFA) for Pt2 and Pt24, respectively, confirming the non-specific incorporation of SeMet into plasma SELENOP after administration (red line). The identification of SeMet in the samples was conducted via spiking experiments with a SeMet standard (dotted lines in Fig. 5 and Figure S3b, ESI) and using a second separation method based on reversed-phase HPLC using 0.2% FA. Moreover, the levels of SeMet found for the baseline samples were comparable to those in the SRM 1950 human plasma (control or non-treated sample, data not shown).

The incorporated SeMet in the administered samples (Pt2 v4 and Pt24 v4) represented approximately 38% and 47% of the total Se bound to SELENOP, corresponding to (152.3 ± 1.4) µg kg^−1^ Se and (144.9 ± 2.9) µg kg^−1^ Se, respectively. Therefore, after SeMet administration, Se bound to SELENOP increased up to 85% (Table 2), and approximately 45% of this increase appears to be attributed to the non-specific incorporation of SeMet into the SELENOP affinity fraction. It is also noteworthy that changes were only observed in the ratios SELENOP/Se and Se-ALB/Se after SeMet administration (0.6 and 0.3, respectively), whilst they remained constant for SS and MSC (0.7 and 0.2, values also shown at baseline). This could potentially explain the significant increase in plasma SELENOP concentration with SeMet (~ 85%) compared to SS or MSC (~ 30%). SeMet seems to participate in two different Se pools (the non-regulated Met pool and the regulated one), while SS and MSC may only contribute to the regulated Se metabolism (mainly due to SELENOP and maybe other selenoproteins).

## Conclusions

A multi-Se compound response comparison of plasma Se biomarkers in cancer patients is presented for the first time by elemental and speciation studies. The major plasma biomarkers of Se status (total Se, selenoproteins, and LMW Se species) were accurately quantified in plasma from cancer patients before and after treatment with the three Se compounds (SS, SeMet, and MSC) administered at 400 µg Se/day for 28 days (Table 3).

**Table 3:** Selenium biomarker analysis in cancer patients after Se administration. Mean values of the major plasma biomarkers of Se status (detailed in Fig. 1) before (v2) and after treatment (v4), expressed as µg kg^−1^ Se (mean ± expanded uncertainty, 2 independent measurements per sample, *k* = 2, *n* = 23)

Type of analysis	Se biomarker	Mean, µg kg^−1^ Se
Total Se in plasma (Fig. 1a)	Total Se v2	93.7 ± 8.7
	Total Se v4	147.7 ± 21.7
Se speciation in plasma (Fig. 1b)	ΣSe species v2	97.6 ± 8.1
	ΣSe species v4	154.4 ± 22.1
	Se-SELENOP v2	65.3 ± 5.5
	Se-SELENOP v4	94.6 ± 10.2
	Se-ALB v2	18.8 ± 2.4
	Se-ALB v4	35.7 ± 8.6
	GPX3 + non-retained Se species v2	13.5 ± 1.4
	GPX3 + non-retained Se species v4	24.2 ± 4.4
Total Se in plasma proteins (Fig. 1c)	Total Se HMW v2	92.2 ± 6.6
	Total Se HMW v4	148.1 ± 19.3
	Total Se LMW_calculated_ v2	5.2 ± 2.7
	Total Se LMW_calculated_ v4	7.6 ± 4.2
	§GPX3_calculated_ v2	8.8 ± 0.9
	§GPX3_calculated_ v4	18.7 ± 2.2
Speciation of LMW Se species (Fig. 1c)	ΣLMW v2	0.35 ± 0.19
	ΣLMW v4	3.35 ± 1.39
	Selenosugar-1 v2	< LOQ
	Selenosugar-1 v4	1.16 ± 0.30
	MSC v2	< LOQ
	MSC v4	1.61 ± 1.75
	Non-retained LMW Se species v2	0.35 ± 0.19
	Non-retained LMW Se species v4	1.35 ± 0.38

^§^ GPX3_calculated_ = Total Se HMW—(Se-SELENOP + Se-Alb)

A relatively small impact of Se administration on the LMW Se pool (≤ 5%) was observed for all patients after treatment, being selenosugar-1 the most relevant LMW Se species particularly after administration of MSC (*p* = 1.3 × 10^−4^). Therefore, special attention was paid to the HMW Se pool. In this pool, SELENOP, Se-ALB, and GPX3 levels increased in correlation with the increase of total Se after the administration with the three Se compounds, and significant differences were obtained between baseline and treated samples. The positive correlation between total Se and the HMW Se biomarkers indicates that subjects were Se-deficient before administration (baseline mean plasma Se levels of 93.7 ± 8.7 µg kg^−1^ Se) and that full expression of selenoproteins was not achieved at ~ 400 µg Se/day, pointing out the need for higher Se doses or a longer period of administration. While a moderate Se increase (~ 30%) was found for SS and MSC, SeMet administration increased the Se levels up to 85% for Se bound to SELENOP.

For the first time, evidence has been provided here for the in vivo non-specific incorporation of Se into the SELENOP fraction (accounting for 45% of the total Se increase) when SeMet was administered. These findings are in line with the involvement of SeMet in both the Met and regulated Se pools, while other forms of Se only contribute to the saturable pool. This dual pathway of SeMet could offer unique advantages compared to other Se forms that predominantly contribute to only one pool, such as longer body retention time and a greater protein reservoir for SeMet. However, it is worth noting that the effects of Se administration can vary depending on the chemical form, dose, and duration of treatment, as well as individual factors such as baseline Se status and the presence of specific health conditions.

The insights into the mechanisms underlying the effects of administration of these Se compounds provided by this study will be further used to support a further trial in which Se forms will be combined with chemotherapy drugs aiming to achieve an optimal therapy. In other words, these results, and those from further evaluation at higher Se doses, have the potential to help define the optimal Se dose and compound for use in future randomised clinical trials with cancer therapies [10, 11]. Future work will include the determination of methylselenol, a critical Se metabolite that mediates its chemopreventive effect as well as the therapeutic interactions of Se compounds with cancer treatments.

### Supplementary information

Below is the link to the electronic supplementary material.

Supplementary file1 (PDF 0.99 MB)
