# Supplementary material for: Selenium speciation studies in cancer patients to evaluate the responses of biomarkers of selenium status to different selenium compounds
Source: Anal Bioanal Chem. 2024 Jan 30;416(11):2835–48. doi: 10.1007/s00216-024-05141-y (PMC11009772; doi:10.1007/s00216-024-05141-y)
Supplement: Supplementary file 1 — Supplementary file1 (PDF 0.99 MB) [file 216_2024_5141_MOESM1_ESM.pdf]

ELECTRONIC SUPPLEMENTARY INFORMATION (ESI)

SELENIUM SPECIATION STUDIES IN CANCER PATIENTS TO EVALUATE THE RESPONSES OF BIOMARKERS OF SELENIUM STATUS TO DIFFERENT SELENIUM COMPOUNDS

M. Estela del Castillo Busto[1,2,*], Christian Ward-Deitrich[1], Stephen O. Evans[3,4], Margaret P. Rayman[5], Michael B. Jameson[4,6] and Heidi Goenaga-Infante[1,*]

[1]LGC Limited, National Measurement Laboratory (NML), Queens Road, Teddington, Middlesex TW11 0LY, United Kingdom

[2]Grupo Química Analítica Aplicada (QANAP), Instituto Universitario de Medio Ambiente (IUMA), Universidade da Coruña (UDC), 15071 A Coruña, Spain

[3]Department of Biological Sciences, University of Waikato, Hamilton, New Zealand

[4]Waikato Clinical Campus, University of Auckland, Hamilton, New Zealand

[5]Department of Nutritional Sciences, University of Surrey, Guildford GU2 7XH, United Kingdom

[6]Oncology Department, Waikato Hospital, Hamilton, New Zealand

* Corresponding authors' email: M. Estela del Castillo Busto, estela.delcastillo@udc.es; Heidi Goenaga-Infante, Heidi.Goenaga-Infante@lgcroup.com

Table S1. Operational conditions of ICP-MS/MS and HPLC-ICP-MS/MS

General ICP-MS Conditions: *Agilent 8800 ICP-MS/MS*	
RF power	1550 W
Plasma gas flow	15 L min^{-1}
Auxiliary gas flow	0.9 L min^{-1}
Carrier gas flow	0.9 - 1.17 L min^{-1}
Cell gas flow rate/settings	40 – 48 % O_2, 1.2 - 1.5 mL min^{-1} H_2
Measurement Conditions	
Total Se analysis-	Spectrum mode, ICP-MS/MS
Points per peak	3
Replicates	5
Sweeps/Replicate	100
Measured *m/z*	$^{77}Se \rightarrow ^{93}SeO$, $^{78}Se \rightarrow ^{94}SeO$, $^{80}Se \rightarrow ^{96}SeO$
	IS: $^{73}Ge \rightarrow ^{73}Ge$, $^{103}Rh \rightarrow ^{119}RhO$
Se speciation analysis-	Time resolved analysis (TRA), HPLC-ICP-MS/MS
Measured *m/z*	$^{77}Se \rightarrow ^{93}SeO$, $^{78}Se \rightarrow ^{94}SeO$, $^{80}Se \rightarrow ^{96}SeO$
	IS: $^{103}Rh \rightarrow ^{119}RhO$ (only for RP-IP-HPLC-ICP-MS/MS)
Integration time	0.3 s / mass, 0.1 s / mass (IS)
Double Affinity HPLC conditions	
Affinity columns	HiTrap® Heparin and HiTrap® Blue HP (1 mL)
Binding Buffer (A)	0.05 mol L^{-1} Ammonium Acetate, pH 7.0
Eluting Buffer (B)	1.5 mol L^{-1} Ammonium Acetate, pH 7.0
Flow rate	0.5 mL min^{-1}
Injection volume	50 µL, 4 °C
Gradient	0 - 10 min, A, Position P1 (load)
	10.01 - 30 min, B, Position P2 (inject)
	30.01 - 35 min, B, Position P1
Run time	10 min (standards), 35 min (samples)
Reversed-phase (RP) HPLC conditions	
RP column	Agilent Zorbax C_8 column (250 x 4.6 mm i.d.; 5 µm)
Mobile phase	Aqueous 2 % (v/v) MeOH with 0.1 % (v/v) TFA, for IP-RP-HPLC
	Aqueous 2 % (v/v) MeOH with 0.2 % (v/v) FA, for RP-HPLC
Flow rate	1.0 mL min^{-1}, isocratic
Injection volume	50 µL, 4 °C
Run time	25 min

Table S2. Total plasma Se concentrations by double AF-ICP-MS at baseline (v2) and after 4 weeks of administration (v4) expressed as μg kg^{-1} Se: mean ± expanded uncertainty (2 independent measurements per sample, *k*=2), median, interquartile range (IQR) in all study subjects (n = 23) and in the randomised groups (SS, SeMet and MSC). Results of the comparison of the two-time points expressed as increase of Se (ΔSe, %) and p-value (paired t-test). And results of Pearson correlation analysis (correlation coefficient, r) between total Se by ICP-MS (**Table 1**) and double AF-ICP-MS. $p < 0.01$ (**), and $p < 0.001$ (***).

	**Total plasma Se by speciation (double AF-ICP-MS)**					
**Sample**	**Mean**	**Median**	**IQR**	**ΔSe, %**	**p-value**	**r**
v2, n=23	97.6 ± 8.1	96.0	88.6-103.3	61 ± 12	1.7 10^{-5} (***)	0.9
v4, n=23	154.4 ± 22.1	134.6	119.4-172.0			1.0
SS v2, n=8	97.9 ± 11.6	98.2	90.6-105.1	27 ± 6	2.0 10^{-3} (**)	0.9
SS v4, n=8	122.7 ± 12.8	124.2	112.2-128.6			0.9
SeMet v2, n=7	96.4 ± 17.5	99.5	88.5-101.4	130 ± 19	2.9 10^{-4} (***)	0.9
SeMet v4, n=7	216.6 ± 35.1	216.4	188.6-250.3			0.9
MSC v2, n=8	98.4 ± 12.9	91.7	90.1-97.9	35 ± 6	2.5 10^{-4} (***)	0.9
MSC v4, n=8	131.7 ± 15.9	126.3	118.0-139.6			0.9

Table S3. Total Se concentrations in the HMW ultrafiltered plasma fractions by ICP-MS at baseline (v2) and after 4 weeks of administration (v4) expressed as µg kg^{-1} Se: mean ± expanded uncertainty (2 independent measurements per sample, *k*=2), median, interquartile range (IQR) in all study subjects (n = 23) and in the randomised groups (SS, SeMet and MSC). Results of the comparison of the two-time points expressed as increase of Se (ΔSe, %) and p-value (paired t-test). And results of Pearson correlation analysis (correlation coefficient, r) between total Se in the whole plasma by ICP-MS (**Table 1**) and in the HMW ultrafiltration one. $LMW_{calculated}$ is calculated indirectly by the subtraction of Se in the HMW pool from total plasma Se, expressed as percentage relative to total content. $p < 0.01$ (**), and $p < 0.001$ (***).

	Total Se in HMW fraction						
**Sample**	**Mean**	**Median**	**IQR**	**ΔSe, %**	**p-value**	**r**	**$LMW_{calculated, \%}$**
v2, n=23	92.2 ± 6.6	88.4	81.9-101.8	64 ± 12	7.0 10^{-6} (***)	0.7	5.5 ± 0.3
v4, n=23	148.1 ± 19.3	135.3	117.5-175.2			0.9	5.1 ± 0.3
SS v2, n=8	90.6 ± 9.5	86.6	81.9-101.5	34 ± 7	1.1 10^{-3} (**)	0.6	7.3 ± 1.3
SS v4, n=8	119.8 ± 11.9	119.0	105.1-132.2			0.8	3.0 ± 2.8
SeMet v2, n=7	92.0 ± 13.8	88.5	84.4-98.7	127 ± 24	8.2 10^{-4} (***)	0.6	4.8 ± 1.1
SeMet v4, n=7	201.0 ± 28.6	190.2	175.2-225.8			0.8	5.7 ± 1.6
MSC v2, n=8	93.9 ± 12.1	90.6	82.0-101.4	38 ± 7	1.7 10^{-3} (***)	0.9	4.3 ± 1.5
MSC v4, n=8	130.0 ± 22.8	123.6	115.2-136.4			0.5	6.4 ± 1.2

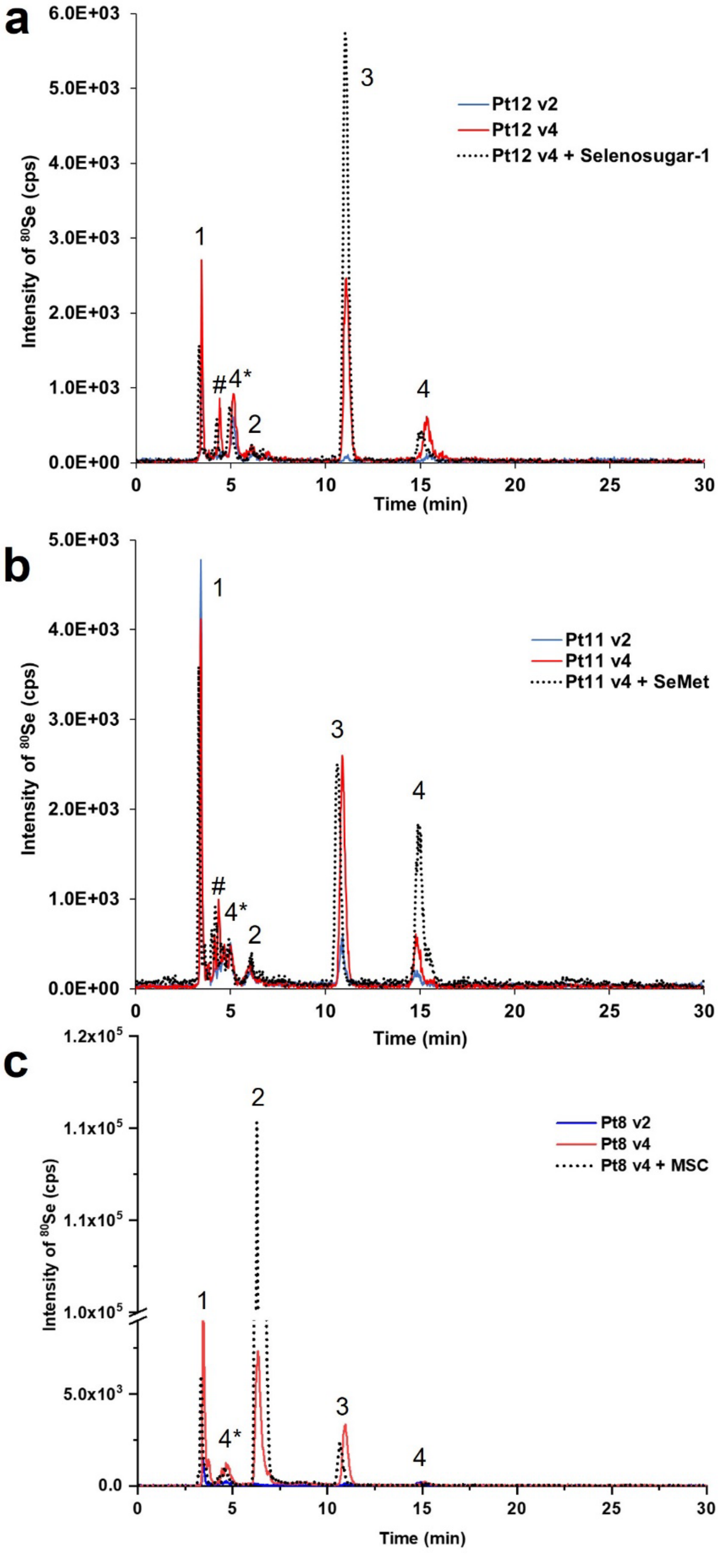

Fig S1. IP-RP-ICP-MS chromatograms of LMW plasma fractions of a) Patient 12 (Pt12), b) Patient 11 (Pt11) and c) Patient 8 (Pt8). Blue line: baseline sample, red line: sample after treatment and doted line: sample after treatment spiked with selenosugar-1, SeMet and MSC, respectively. Peak identification: 1.Non-retained Se, 2.MSC, 3.Selenosugar-1, 4.SeMet and 4*.SeMet oxidised product and #.Unknown.

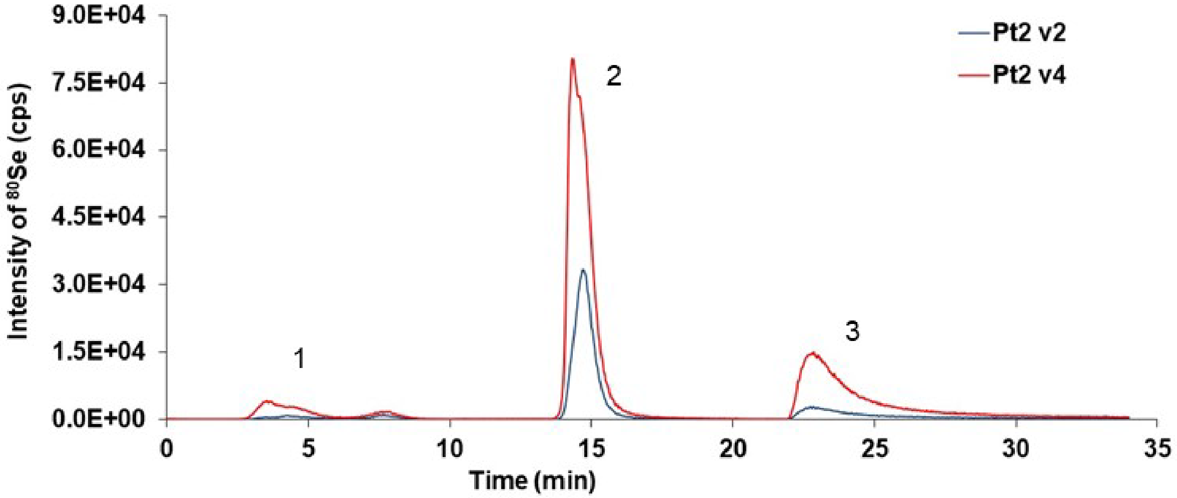

Fig S2. Double AF-HPLC-ICP-MS Se chromatograms of plasma samples from Patient 2 (Pt2). Blue line: baseline sample, red line: sample after treatment. Peak identification: 1.GPX3 + non-retained Se-species, 2.SELENOP and 3.Se-ALB.

Table S4. Validation of the double-AF-ICP-MS Se speciation methodology by the analysis of the NIST SRM 1950 human plasma. Mean, expanded uncertainty U (expressed as µg kg^{-1} Se, k=2) and relative expanded uncertainty U_r (%, k=2) of the different Se-species measured during 4 independent batches (at least 4 injections per batch). Expected IDA values and corresponding obtained recovery (%).

**NIST SRM 1950**	**GP3x + non-retained species**	**SELENOP**	**Se-ALB**	**∑Se-species**
Mean ± U	15.5 ± 3.7	62.6 ± 8.3	21.3 ± 5.4	99.4 ± 17.5
U_r	24	13	24	18
Expected Mean ± U	15.1 ± 1.7[a]	60.6 ± 3.2[b]	23.1 ± 3.7[a]	105.5 ± 3.8[c]
U_r	11	5	16	4
Recovery	102.9	103.3	92.0	94.2

[a] Values obtained by post-column IDA-ICP-MS (16)
[b] Value obtained by double species-specific IDA in combination with HPLC-ICP-MS/MS at the peptide level (26)
[c] Reference value determined by IDA-ICP-MS by NIST (k=2.2)

Table S5. Levels of Se bound to SeALB obtained by double AF-ICP-MS at baseline (v2) and after 4 weeks of administration (v4) expressed as μg kg^{-1} Se: mean ± expanded uncertainty (2 independent measurements per sample, *k*=2), median, interquartile range (IQR) in all study subjects (n = 23) and in the randomised groups (SS, SeMet and MSC). Results of the comparison of the two-time points expressed as increase of Se (ΔSe, %) and p-value (paired t-test). And results of Pearson correlation analysis (correlation coefficient, r) between total Se by ICP-MS (**Table 1**) and double AF-ICP-MS. $p < 0.01$ (**), and $p < 0.001$ (***).

	Se bound to SeALB					
**Sample**	**Mean**	**Median**	**IQR**	**ΔSe, %**	**p-value**	**r**
v2, n=23	18.8 ± 2.4	18.1	15.5-23.0	17 ± 4	$7.0\ 10^{-4}$ (***)	0.7
v4, n=23	35.7 ± 8.6	25.4	22.1-47.4			0.9
SS v2, n=8	18.9 ± 3.0	17.1	15.8-23.5	23 ± 5	$1.4\ 10^{-3}$ (**)	0.5
SS v4, n=8	22.8 ± 2.6	22.3	21.1-24.1			0.8
SeMet v2, n=7	16.7 ± 4.0	15.6	13.7-17.8	288 ± 30	$6.8\ 10^{-5}$ (***)	0.8
SeMet v4, n=7	62.1 ± 10.7	62.5	52.7-69.2			0.8
MSC v2, n=8	20.7 ± 4.9	19.3	17.6-22.8	26 ± 7	$3.8\ 10^{-3}$ (**)	0.7
MSC v4, n=8	25.4 ± 5.6	23.7	22.0-26.5			0.5

Table S6. Levels of Se bound to GPX3 obtained by double AF-ICP-MS at baseline (v2) and after 4 weeks of administration (v4) expressed as $\mu g\ kg^{-1}$ Se: mean ± expanded uncertainty (2 independent measurements per sample, *k*=2), median, interquartile range (IQR) in all study subjects (n = 23) and in the randomised groups (SS, SeMet and MSC). Results of the comparison of the two-time points expressed as increase of Se (ΔSe, %) and p-value (paired t-test). And results of Pearson correlation analysis (correlation coefficient, r) between total Se by ICP-MS (**Table 1**) and double AF-ICP-MS. $p < 0.01$ (**), and $p < 0.001$ (***).

	Se bound to GPX3					
**Sample**	**Mean**	**Median**	**IQR**	**ΔSe, %**	**p-value**	**r**
v2, n=23	8.8 ± 0.9	6.5	2.1-13.7	5.1 ± 1.4	$3.3\ 10^{-2}$	-0.3
v4, n=23	18.7 ± 2.2	15.0	4.4-24.3			0.1
SS v2, n=8	6.6 ± 0.5	6.7	0.9-8.2	5.9 ± 2.3	$7.8\ 10^{-2}$	-0.7
SS v4, n=8	14.5 ± 1.4	15.4	11.1-16.7			-0.1
SeMet v2, n=7	10.7 ± 1.1	6.5	4.3-13.7	2.4 ± 0.8	$3.5\ 10^{-1}$	-0.4
SeMet v4, n=7	21.2 ± 1.8	5.9	1.4-37.1			-0.1
MSC v2, n=8	9.4 ± 0.9	4.7	2.7-17.6	6.8 ± 3.1	$2.3\ 10^{-1}$	0.1
MSC v4, n=8	20.7 ± 2.9	15.9	11.4-24.0			0.3

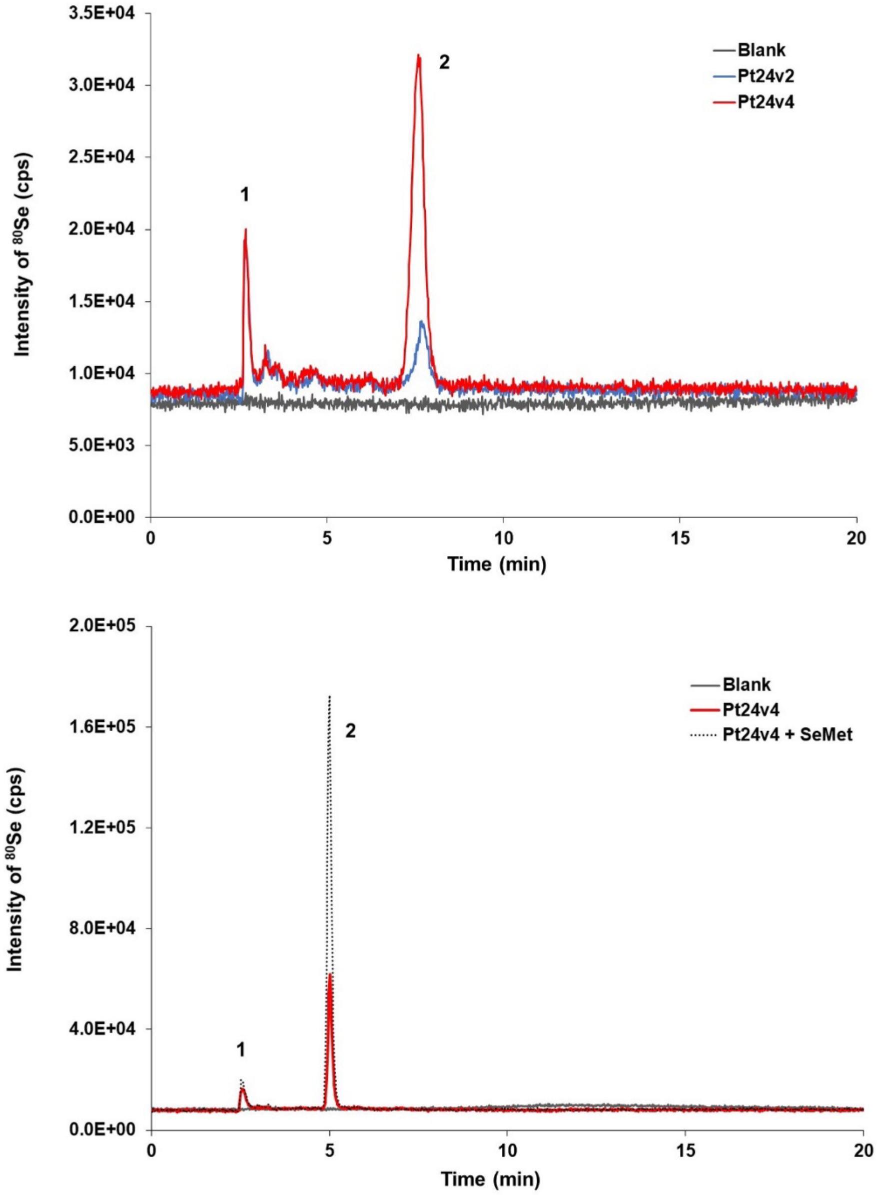

Fig S3. Determination of SeMet released from the enzymatic extraction of SELENOP affinity plasma fractions from Patient 24 (administrated with SeMet). A) IP-RP-ICP-MS chromatograms (0.1 % TFA, Table S1) of Pt24 before (Pt24v2) and after (Pt24v4) SeMet administration. B) RP-ICP-MS chromatograms (0.2 % FA, Table S1) of Pt24v4 spiked with organic SeMet for identification. Peak identification: 1.Non-retained Se, 2.SeMet.
